# Wnt Signalling in Intestinal Stem Cells: Lessons from Mice and Flies

**DOI:** 10.3390/genes9030138

**Published:** 2018-03-02

**Authors:** Jessica Perochon, Lynsey R. Carroll, Julia B. Cordero

**Affiliations:** 1 Wolfson Wohl Cancer Research Centre, Institute of Cancer Sciences, University of Glasgow, Garscube Estate, Switchback Road, Glasgow G61 1QH, UK; Jessica.Perochon@glasgow.ac.uk (J.P.); l.carroll.1@research.gla.ac.uk (L.R.C.); 2 CRUK Beatson Institute, Institute of Cancer Sciences, University of Glasgow, Garscube Estate, Switchback Road, Glasgow G61 1BD, UK

**Keywords:** Wnt signalling, intestine, homeostasis, regeneration, stem cells, *Drosophila*, mouse models, cancer

## Abstract

Adult stem cells play critical roles in the basal maintenance of tissue integrity, also known as homeostasis, and in tissue regeneration following damage. The highly conserved Wnt signalling pathway is a key regulator of stem cell fate. In the gastrointestinal tract, Wnt signalling activation drives homeostasis and damage-induced repair. Additionally, deregulated Wnt signalling is a common hallmark of age-associated tissue dysfunction and cancer. Studies using mouse and fruit fly models have greatly improved our understanding of the functional contribution of the Wnt signalling pathway in adult intestinal biology. Here, we summarize the latest knowledge acquired from mouse and *Drosophila* research regarding canonical Wnt signalling and its key functions during stem cell driven intestinal homeostasis, regeneration, ageing and cancer.

## 1. Introduction

Wingless-related integration site (Wnt) was named after the *Drosophila wingless* gene and the mouse *int1* gene. *Int1* was discovered in 1982 as a gene overexpressed in breast cancer [1]. Subsequent studies revealed first the amino acid sequence and next demonstrated that *Int1* was a secreted protein with the potential to act as a signalling molecule [2,3]. Five years later, *int1* was found to be homolog to the *Drosophila* gene *wingless* (*wg*), which had been previously characterized as a segment polarity gene through seminal work that identified regulators of body axis during embryonic development [4,5]. Thereafter, *int*/*wingless* became Wnt, giving also the generic name to the pathway itself, and *Int1* became Wnt1 as the first ligand identified. More than three decades later, there are no doubts about the importance of the Wnt pathway as an evolutionarily conserved system, which is broadly implicated in diverse biological processes such as embryonic development, adult tissue homeostasis, regeneration and disease [6,7,8].

The Wnt pathway is divided into β-catenin-dependent (canonical) and independent (non-canonical) signalling. In both cases, the major components of the pathway are the Wnt ligands, which act in an autocrine or paracrine fashion by binding their Frizzled (Fz) receptors. In canonical Wnt signalling, Fz receptors engage with co-receptors Lrp5/6, at the cell surface. Here, we will focus on the canonical pathway, as it is the one mainly studied in stem cell biology and the intestine. Briefly, in steady state conditions, the levels of cytoplasmic β-catenin are kept low through phosphorylation by a complex of proteins, known as the ‘destruction complex’, which includes Axin, Adenomatous polyposis coli (Apc), glycogen synthase kinase 3 (Gsk3) and casein kinase 1α (Ck1α) [9]. This destruction complex promotes the ubiquitination of β-catenin and its degradation by the proteasome. Moreover, the ADP-ribose polymerase Tankyrase (Tnks) has been described to target Axin and stimulate its degradation through proteolysis [10]. Activation of the signalling pathway upon binding of Wnt ligands to receptors initiates a series of signalling events, including the activation by phosphorylation of cytoplasmic Dishevelled (Dsh), which ultimately leads to inactivation of the destruction complex and stabilization of β-catenin, its accumulation in the cytosol and translocation into the nucleus where it forms complexes with Tcf/Lef transcription factors, among others, to regulate target gene expression [8,11,12].

Work in the last two decades has demonstrated a central role of the Wnt pathway in the regulation of adult stem cells and, hence, the maintenance of tissue homeostasis. Stem cells are highly dependent on extrinsic cues derived from their microenvironment, also known as niche. Wnt signals are an essential component of a wide range of stem cell niches, including that of the gastrointestinal epithelium [13,14].

The intestinal epithelium is constantly turned over through the action of dedicated intestinal stem cells (ISCs). This process needs to be sustained and highly regulated as its disruption leads to either tissue wasting or the development of gastrointestinal disorders, including cancer. In this review, we will summarize the latest findings regarding the contribution of canonical Wnt signalling to ISCs and their activity during normal tissue homeostasis, regeneration, ageing and intestinal cancer. We will discuss data derived from studies in mice and the fruit fly *Drosophila melanogaster*, with an emphasis on the use of the fly as an increasingly valuable model system and powerful genetic tool for studying various aspects of Wnt signalling in intestinal health and pathogenesis.

## 2. The Adult Mammalian Intestine

**Figure 1:**
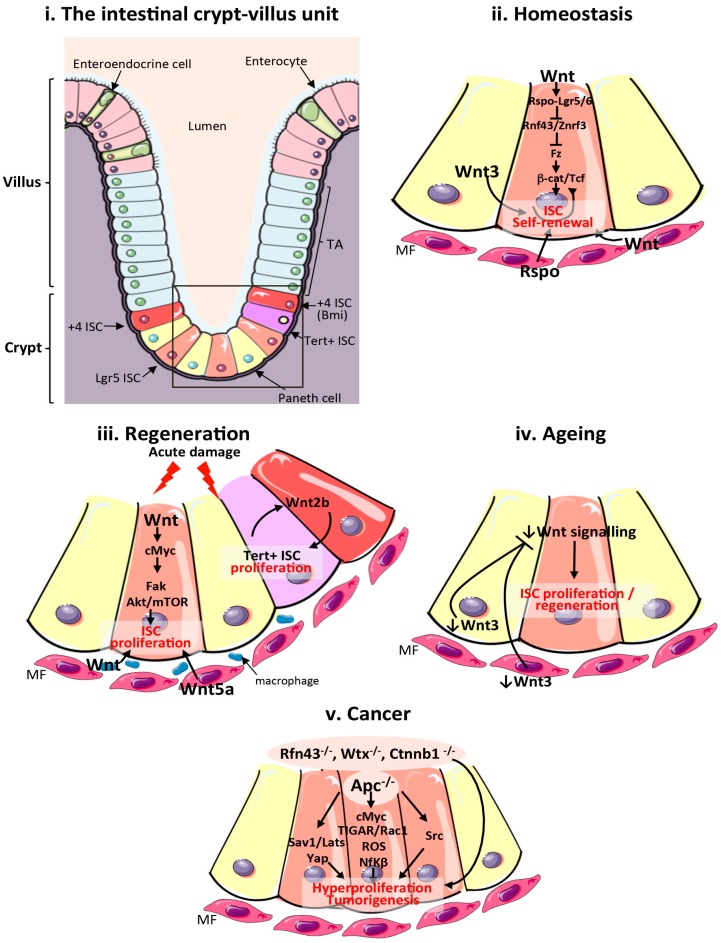
Wnt signalling in the mammalian intestine during homeostasis, regeneration, ageing and cancer. (**i**) Schematic of the cellular composition and architecture of a crypt–villi unit in the mammalian intestine. The stroma is depicted in purple and the gut lumen in peach. The boxed area highlights the crypt and stem cell niche, which are magnified in (**ii**–**v**); (**ii**) Main sources of the Wnt stem cell niche and pathway activation during intestinal homeostasis. Wnt3 from Paneth cells, and Wnt and Rspo from mesenchymal and epithelial stem cell niches are important sources of Wnt during homeostasis; (**iii**) Sources of the Wnt stem cell niche and pathway activation during intestinal regeneration following damage. Wnt2b signalling from intestinal epithelial cells is required to activate proliferation of quiescent Tert + intestinal stem cells (ISCs). Wnt from macrophages and Wnt5a from the mesenchyme are also important sources of Wnt during regeneration. Wnt activation of cMyc is known to target Fak and Akt/mTOR pathways to increase ISC proliferation in response to damage; (**iv**) Reduced production of mesenchymal and Paneth cell Wnt3 and canonical Wnt pathway activity in the ageing intestinal epithelium impairs ISC proliferation; (**v**). Wnt pathway activation during intestinal tumourigenesis and functional pathways activated following Apc loss. ISCs: intestinal stem cells; TA: transit amplifying cells; Tert: telomerase reverse transcriptase; MF: mesenchymal fibroblasts; Apc: Adenomatous polyposis coli; Fak: focal adhesion kinase; ROS: reactive oxygen species; TIGAR: TP53-inducible glycolysis and apoptosis regulator; Rspo: R-Spondin; Sav1: Salvador 1; mTOR: mammalian target of rapamycin; Yap1: Yes associated protein 1; Lats: Large tumour suppressor kinase 1; Nf-κβ: Nuclear factor κβ.

The mammalian gut develops from the endoderm. During gastrulation the undifferentiated endoderm is pre-patterned into three regions along the anterior–posterior axis: the foregut, which forms the stomach and other organs; the midgut, which forms the small intestine; and the hindgut, which gives rise to the large intestine [15]. The adult gastrointestinal tract is a tubular structure composed of three layers consisting of smooth muscle, connective tissue and mucosa. The intestinal epithelium is part of the mucosa layer, which is supported by the lamina propia and the muscularis mucosae. The mucosa acts as a barrier, preventing the entry of harmful substances and exhibits both innate and adaptive immune functions. It also acts as a selective filter, which enables the uptake of nutrients, water, and various other beneficial materials from the intestinal lumen. From anterior to posterior the mammalian intestinal tube is comprised of the small intestine, which is divided into duodenum, jejunum, and ileum, and the large intestine, which consists of the cecum, colon and rectum. The architecture of the small intestine is organised in a way that maximises the surface area available for the absorption of nutrients. This is due to the presence of crypts of Lieberkühn and protrusions of the epithelia known as villi (Figure 1i). The large intestine comprises a simple columnar epithelium with crypts of Lieberkühn and a proliferative stem/progenitor zone. However, unlike the small intestine, the large intestine lacks villi [16].

The mammalian intestine contains multipotent stem cells, which have the ability to self-renew and generate undifferentiated transit amplifying cells and the various specialized cell types within the intestinal epithelium, such as the absorptive enterocytes, secretory goblet cells, enteroendocrine cells and Paneth cells (Figure 1i). One of the most notable advancements in the field of intestinal biology has been the discovery of various populations of stem cells within the intestine, characterized by the expression of specific markers, such as Lgr5 [17], Bmi1 [18], Musashi1 (Msi1) [19], Ascl2 [20] and Sox9 [21] among others. Lgr5 is perhaps the most common marker used to target, functionally characterize and lineage trace mammalian ISCs [17]. Lgr5 belongs to the G protein coupled receptor family and it is one of 80 known Wnt target genes in the mammalian intestine [22,23]. ISCs were first discovered using labeling with [^3^H] Thymidine following irradiation-induced damage of the tissue [24,25,26]. Studies using this method were able to define the label retaining +4 position crypt cells as a population of slowly cycling stem cells, which are distinct from the fast-cycling stem cells, also called crypt base columnar cells (CBC) (Figure 1i). Crypt base columnar cells show active proliferation in response to homeostatic niche signals, while label-retaining cells remain quiescent during normal homeostasis and proliferate in response to damage.

Fast-cycling stem cells have generally been distinctly identified by their expression of the *lgr5*, *Olfm4* and *Ascl2* genes, while slower cycling stem cells have been characterized by the expression of markers including Bmi1, Lrig1, Hopx and telomerase reverse transcriptase (mTert) [18,27,28,29,30,31,32]. Nevertheless, recent studies using single cell RNA sequencing technology have revealed subpopulations of cells that exhibit properties of both slow and fast cycling stem cells [33,34,35]. A subpopulation of label-retaining cells acts as Lgr5 precursor cells that can regenerate the tissue upon damage [34], while a subpopulation of Lgr5 stem cells co-expressing Mex3a also act as a slowly cycling reserve pool of stem cells able to rapidly divide upon damage [35]. More work is needed to elucidate the role and regulation of these cell populations.

Intestinal stem cells reside at the base of the crypt while most differentiated cells migrate up the crypt–villus axis in the small intestine [36] with the exception of Paneth cells, an essential component of the intestinal stem cell niche [37], which intercalate between ISCs at the crypt base (Figure 1i). The distinct compartmentalization of stem cells from their differentiated linages, especially in the small intestine, has been attributed to gradients of various signalling pathways along the crypt–villus axis, including Wnt and EphB [22,38,39]. Β-catenin and Tcf inversely control the expression of EphB2 and EphB3 receptors and their ligand Ephrin-B1 within the crypt–villus axis, which leads to higher Eph signalling further from the crypt base. Experiments using *EphB2*/*EphB3* null mice showed that these genes are required to restrict cell intermingling and compartmentalize cell populations within the intestinal epithelium [40].

## 3. The Adult *Drosophila* Intestine

The adult digestive tract of the fruit fly *Drosophila melanogaster* is a tubular structure surrounded by visceral muscle, enteric neurons and gut-associated trachea, which are akin to the mammalian vasculature. As it is the case for the mammalian intestine, the fly gut ensures essential physiological functions of the living organism, such as the incorporation and processing of food, nutrient absorption and elimination of solid waste, and displays key endocrine, immune and metabolic roles. The fly intestine consists of a monolayer epithelium, divided into three domains of different developmental origins: the foregut, the midgut, and the hindgut. The foregut and the hindgut epithelium are of ectodermal origin whereas the midgut epithelium originates from the endoderm. The foregut comprises the pharynx, the esophagus and the crop. The midgut extends from the cardia until the junction with the hindgut, where the Malpighian tubules, which display functions similar to the mammalian kidneys, connect with the gut. The *Drosophila* adult midgut is described to be the structural and functional equivalent of the mammalian small intestine [17,41].

**Figure 2:**
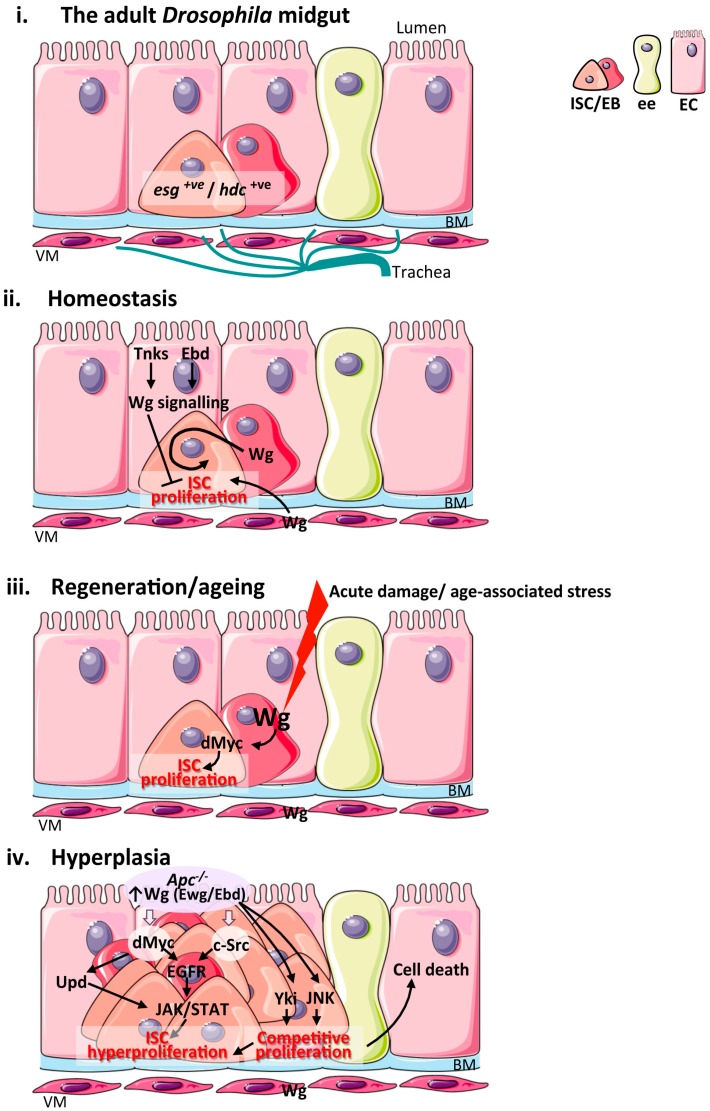
Wnt signalling in the adult *Drosophila* midgut during homeostasis, regeneration, ageing and hyperplasia. (**i**) Schematic of the cellular composition and architecture of the adult *Drosophila* midgut epithelium and its microenvironment; (**ii**) Main sources of the Wnt stem cell niche and pathway activation during intestinal homeostasis. The VM and EBs produce Wg ligand to active signalling within ISC and drive their proliferation. Wg signalling activation in ECs by Tnks and Ebd inhibit ISC proliferation non-autonomously; (**iii**) Sources of the Wg stem cell niche and pathway activation during intestinal regeneration following damage and upon ageing. Up-regulation of Wg from EBs activates Wg signalling and its downstream target dMyc to drive ISC proliferation. The VM niche expresses Wg but is dispensable for ISC proliferation in this context; (**iv**) Wnt pathway activation during intestinal hyperplasia and functional pathways downstream of *Apc*. JNK and Yki activation in *Apc*^−/−^ cells drive ISC proliferation and cell competition leading to apoptosis of neighbouring wild type cells. ISC: intestinal stem cell; EB: enteroblast; EC: enterocyte; ee: enteroendocrine cell; BM: basement membrane; VM: visceral muscle; *esg*: *escargot*; *hdc*: *headase*; Wg: Wingless; Tnks: Tankyrase; Ebd: Earthbound; Ewg: Erect wing; Yki: Yorkie; JNK: c-Jun N-terminal kinase; Upds: Unpaired cytokines; EGFR: Epithelial growth factor receptor; JAK: Janus kinase.

The fly midgut is replenished by ISCs [42,43]. *Drosophila* ISCs undergo cell division to renew themselves and generate uncommitted enteroblasts (EBs), which are progenitor cells that can further differentiate into either secretory enteroendocrine cells (EEs) or absorptive enterocytes (ECs) [44]. ISCs and EBs are characterized by the expression of the snail family transcription factor *escargot* (*esg*) [42] and *headcase* (*hdc*) [45]. *Drosophila* ISCs do not reside within discrete anatomical locations equivalent to the mammalian crypts and are instead scattered along the basal membrane of the intestinal epithelium. However, they are in either direct or close contact with their microenvironment, which includes uncommitted progenitor cells (EBs), differentiated midgut epithelial cells, visceral muscle (VM) and trachea cells, which all constitute niches as they provide factors that regulate ISC self-renewal and differentiation [46,47,48,49,50,51,52] (Figure 2i).

Due to the similarities between the *Drosophila* midgut and mammalian intestine and the relative simplicity of the invertebrate model system, the adult fly midgut has become a powerful paradigm for investigating the role of many conserved signalling pathways, including Wnt signalling, in the regulation of ISC activity [51,53].

## 4. Wnt Signalling in Mammalian Intestinal Homeostasis and Regeneration

Initial evidence towards a central role of the Wnt pathway in intestinal-stem-cell homeostasis came from pioneering studies in mice showing that genetic ablation of canonical Wnt singling transcription factors Tcf-4 and β-catenin or the use of an inhibitor of the Wnt receptor, Dickkopf-related protein 1 (Dkk1), abolished the proliferative capacity of the small intestine and led to severe disruption of intestinal epithelial integrity, including the loss of crypts [54,55,56,57,58,59].

The discovery of Lgr5 as a stem cell marker [17] and the establishment of intestinal organoids from purified crypts [60] represented a turning point in the study of ISC biology as it permitted, amongst other things, targeted manipulation of genes within ISCs and the isolation of the intestinal epithelium for in vitro studies. Single-sorted Lgr5 positive (Lgr5^+ve^) stem cells were first shown to be able to produce an organised crypt–villus organoid structure in the absence of the epithelial niche [60]. Later studies reported that the efficiency of organoid formation from single Lgr5^+ve^ cells was significantly increased by the addition of Paneth cells due to the provision of a Wnt niche [37]. The importance of an epithelial and mesenchymal Wnt-niche in the maintenance of stem cells in the intestine has been recently challenged by a study reporting that impairment of Wnt secretion from the intestinal epithelium or underlying smooth muscle through conditional knockout of *porcupine* resulted in no obvious defects on intestinal epithelial structure, Wnt activation or the proliferative rate of ISCs [61]. That study suggested a potential redundant nature of the Wnt stem cell niche in the mammalian intestine, which was later confirmed by a recent report revealing intestinal defects following global prevention of Wnt secretion through ubiquitous knockout of Wntless [62].

Lgr receptors potentiate Wnt signalling within ISCs following binding to the ligand, R-spondin. R-spondin/Lgr binding leads to activation of downstream Wnt signalling through downregulation of Fz specific E3 ubiquitin ligases Rnf43 and Znrf3 [63,64] (Figure 1ii). Impairment of Wnt signalling by Rnf43 and Znrf3 suppresses proliferation in the intestine through ubiquitination and degradation of surface-expressed Fz5 [63]. The receptor, Fz7 is also enriched in and required for ISC function in the mammalian intestine [65]. Recent research has demonstrated the importance of a non-redundant cooperation between R-spondin and Wnt signalling, by showing that Wnt proteins are required to prime ISCs by maintaining R-spondin receptor expression, which then drives the further expansion of stem cells via R-spondin ligands [66].

In addition to its function in intestinal homeostasis, Wnt signalling also plays a crucial role during the regeneration of the mammalian intestine following injury. The intestinal epithelium can robustly regenerate in response to multiple forms of stressors/damaging agents that disrupt the tissue, such as cytotoxic drugs [67], gamma radiation [68], and following surgical resection [69]. Regeneration is characterized by an increase in proliferation within the crypt compartment [70]. Multiple studies show Wnt signalling activation as a key event in the induction of intestinal regeneration (Figure 1iii). The Wnt target *c*-*Myc* is upregulated within the crypt compartment of the intestinal epithelium in response to DNA damage and its activation is essential to induce intestinal regeneration through focal adhesion kinase (Fak) and Akt/mTOR signalling [71]. Additionally, *Wnt5a* is expressed by the stroma in response to intestinal damage and induces crypt regeneration in a TGFβ-dependent manner [72]. Interestingly, Wnt produced by macrophages, a key constituent of the stroma, represents an important component of the stem cell niche, which is required for intestinal regeneration and animal survival following damage by irradiation [73]. More recently, damage-inducible Wnt2b expressed in epithelial cells within the crypt has also been proven essential for the regeneration of the intestine upon irradiation by stimulating proliferation of a subpopulation of quiescent Tert^+ve^ ISCs [74] (Figure 1iii). While conditional ablation of Tert^+ve^ cells by diphtheria toxin A expression did not affect intestinal homeostasis, it impaired tissue regeneration following injury [74]. Altogether, this evidence points to the presence of multiple inducible, non-redundant Wnt ligand sources, which are essential for pathway activation and the execution of intestinal regeneration.

## 5. Wnt Signalling in Intestinal Homeostasis and Regeneration in *Drosophila*

In addition to the well-known general benefits offered by their short life cycle and large number of offspring, there are at least two key advantages of using *Drosophila* for the study of Wnt signalling in the intestine: the presence of genetic tools to individually label every cell types within the midgut, and the low redundancy of Wnt ligands.

Multiple-redundant stem cell populations have been identified in mouse models [18,27,28,29,30,31,32,34,35,75]. Moreover, recent studies have highlighted the great plasticity in the mouse intestinal epithelium as evidenced by the de-differentiation potential of committed lineages, including secretory progenitor enterocytes [76], enteroendocrine cells [77] and Paneth cell precursors [78]. This regain of stemness in order to re-populate the mouse intestine upon damage makes the pool of ‘reserve stem cell potential’ even larger and complicates studies of mammalian ISCs.

Functional studies of ISCs in the adult *Drosophila* midgut are less complex. The discovery of *esg* [42] and, more recently, *hdc* [45] as markers of all stem/progenitor cells (ISCs/EBs), has allowed global targeting of this cell population and unambiguous assessment of their role in intestinal homeostasis and regeneration [45,79]. De-differentiation of committed lineages has not been reported in the adult *Drosophila* midgut, suggesting a lower degree of plasticity or reserve stem cell potential in the invertebrate tissue when compared to the mammalian intestine. However, recent studies have reported the existence of cell division without mitotic spindle formation in polyploid ECs, also known as amitosis, and plasticity in the rate of turnover of ECs as a means to maintain intestinal epithelial homeostasis in conditions where stem cell pools are compromised [80,81].

There are multiple sources of Wnt ligands in the mammalian intestine (Table 1) [54,82], which has complicated studies on the homeostatic Wnt stem cell niche [61,83,84,85]. However, low redundancy in the function of Wnt ligands is revealed during intestinal regeneration upon damage [54,74,84]. Whether the differences between Wnt signalling activity in regeneration and homeostasis reflect the activation of a distinct, ‘regeneration specific’ Wnt signature or different levels of signalling activity required for each process remains to be addressed.

The scenario concerning the Wnt stem cell niche in the *Drosophila* midgut appears simpler, even though it shares similarities with its mammalian counterpart. Only Wingless (Wg) and Wnt4 appear to be expressed in the adult fly midgut (Table 1) and Wg is so far the only Wnt ligand reported to have a functional role in the tissue (Table 1). Pioneering studies identified the visceral muscle (VM), which surrounds the intestinal epithelium, as the main source of the Wg stem cell niche in homeostatic conditions [86] (Figure 2ii). Global knockdown of *wg* or intestinal epithelial loss of genes encoding for *fz* and *fz2* receptors prevented homeostatic ISC self-renewal [86]. Further studies have described and characterized novel sources of the Wg stem cell niche and additional roles of the pathway in intestinal homeostasis and regeneration [87,95,96]. In addition to the visceral muscle, epithelial Wg is expressed in the midgut–hindgut junction in homeostatic tissues. Here, Wnt pathway activation appears graded along the length of the adult intestine, peaking at compartment boundaries [96]. Interestingly, work by the same group has demonstrated that Wg pathway activation within ECs impairs ISC proliferation non-autonomously during homeostasis [96,97] (Figure 2ii).

Wg expression by progenitor cells (EBs) is upregulated following damage to the intestinal epithelium [87,95] (Figure 2iii). Cell-specific knockdown experiments have demonstrated that Wg from EBs activates Wnt signalling and induction of the conserved pathway target dMyc within ISCs to drive ISC proliferation upon damage (Figure 2iii). Importantly, while this source of the ligand is essential to drive ISC proliferation and tissue regeneration in response to injury, it is dispensable for homeostatic ISC self-renewal [87]. A strikingly similar phenomenon has been recently described for Wnt2 in the mouse intestine [74] (Figure 1iii).

## 6. Wnt Signalling in Ageing and Tumorigenesis of the Mammalian Intestine

Aging is a complex process, which leads to a decline in tissue integrity and functionality. Ageing affects stem cell function and, therefore, the regenerative capacity of self-renewing tissues. Persistent expression of *Wnt1* within the skin epidermis, which also contains Lgr5^+ve^ stem cells, leads to senescence and exhaustion of the stem cell compartment through the sustained activation of *mTOR*, resulting in a premature ageing phenotype [98]. Conversely, a decrease in canonical Wnt signalling upon ageing results in reduced regenerative potential of the intestine (Figure 1iv). Wnt3 is reduced within stem cells and their niches in the ageing intestine and the addition of the ligand to intestinal organoids of ageing animals can restore ISC function [90]. This reduction of Wnt signalling in the aging intestine has been proposed to represent a protective mechanism to counteract age-associated mutations that could cause intestinal hyperproliferation. However, this also leads to an overall reduced regenerative potential of ISCs upon ageing. Therefore, a full understanding of ageing-specific Wnt signalling events may lead to the design of targeted therapies to prevent age-associated intestinal dysfunction without driving tissue malignancies, such as cancer.

Wnt signalling is perhaps best known for its role as a key driver of intestinal cancer, typically through loss of the negative regulator of the pathway Apc [99,100]. Apc is part of the destruction complex, which counteracts pathway activation by targeting β-catenin for ubiquitination and degradation by the proteasome [101]. Apc and CRC were first linked by the discovery and characterization of familial adenomatous polyposis (FAP) [102,103], an inherited form of CRC characterized by mutations in the *Apc* gene [104]. *Apc* is mutated in 80–90% of hereditary and spontaneous forms of colorectal cancer (CRC) [105]. Over 60% of the mutations within *Apc* occur in the mutation cluster region (MCR) and affect binding to Axin or β-catenin [106], which results in the accumulation of β-catenin and excessive Wnt signalling. Although with lower incidence, CRC can also occur as a result of activating mutations in the *Ctnnb1* gene that encodes for β-catenin. Two independent *Ctnnb1* mutations have been shown to disrupt specific serine/threonine residues within β-catenin, which are normally subject to Gsk-3β phosphorylation and required for subsequent protein degradation, thereby leading to accumulation of β-catenin [106]. Loss of function mutations in components of the Wnt pathway, beyond Apc, have been associated with CRC. Mutations in *Rnf43* were found by whole exome sequencing in 18% of colorectal adenomas, particularly in cases with high microsatellite instability (MSI-H) [107]. The Wilms tumour suppressor (Wtx), a part of the β-catenin destruction complex, [108] is also mutated in colorectal tumours with MSI-H [109]. However, in this article we will focus on CRC driven by *Apc* loss.

The study of *Apc* and its role in CRC was pioneered by the generation of genetically engineered mouse models [110,111]. Conditional loss of function experiments showed that *Apc* is required for cell proliferation and differentiation within the intestinal epithelium, as well as for the migration of cells along the crypt–villus axis [111]. Later work assessing the contribution of ISCs to the generation of intestinal tumours showed that knockdown of *Apc* within Lgr5^+ve^ stem cells leads to rapid intestinal adenoma formation. This work provided the first demonstration of a role of ISCs as the cells of origin in CRC [112]. Complementarily, in vitro work showed that silencing Lgr5 leads to reduced cell proliferation, migration and the tumourigenic potential of colorectal cancer cell lines [113]. Furthermore, high *Lgr5* expression in cells derived from mouse tumours correlates with strong upregulation of Wnt signalling [113]. However, work on genetically engineered mouse models shows that ablation of Lgr5 stem cells within *Apc*-driven adenomas, is not sufficient to affect tumour burden. Therefore, there may be multiple redundant cell populations, including Lgr5^−ve^ ISCs, which contribute to intestinal tumourigenesis [68]. 

Extensive work has been carried out to determine the mechanisms by which *Apc* loss drives proliferation in CRC. For a comprehensive account of the literature on this subject, please see [114,115]. Our goal here is to highlight some key studies on the mechanisms driving intestinal hyperproliferation following *Apc* loss from ISCs, as this is most directly related to the subject of this review (Figure 1v). One such mechanism is mediated by activation of the Rac1 GTPase. *Apc* loss leads to *myc* activation, which is in turn required to activate Rac1, leading to intestinal tumourigenesis via reactive oxygen species (ROS) production and NF-κB signalling [116]. The TP53-inducible glycolysis and apoptosis regulator (TIGAR), a protein involved in glucose metabolism, cooperates with Rac1 to drive proliferation in response to *Apc* loss in the intestine [117] (Figure 1v).

Activation of the non-receptor tyrosine kinase c-Src is increased by up to 15-fold in human CRC [118]. Functional genetic studies in mice and *Drosophila* show that Src is required to induce intestinal tumourigenesis and ISC proliferation following *Apc* loss [119]. Src activation in ISCs is sufficient to drive intestinal hyperplasia, while conditional knock out of *Apc* and *Src* within Lgr5^+ve^ stem cells resulted in reduced tumour burden and increased animal survival [119] (Figure 1v).

Another pathway characterized as a downstream effector of *Apc* loss in the intestine includes the Hippo signalling pathway, a conserved tumour suppressor pathway associated with CRC [120]. The transcription factor Yes-associated protein (Yap), which is normally inactivated by Hippo signalling, is required for the formation of adenomas following loss of *Apc*. In this context, Apc acts as a scaffold for Hippo pathway kinases Salvador (Sav1) and Large tumour suppressor kinase 1 (Lats) to facilitate phosphorylation and subsequent degradation of Yap [121] (Figure 1v). An accompanying article published in this special issue provides a comprehensive review of Wnt and Hippo signalling interactions in the intestine [122].

The above-described studies support the ‘bottom up’ model of CRC, where loss of *Apc* in the crypt/stem cell compartment is required to induce intestinal tumourigenesis. An opposing model of CRC is the ‘top down’ model, which postulates that cells from the villi can also drive intestinal transformation. *Apc* loss from the villi only is not sufficient for the generation of persistent intestinal tumours [112,123]. However, combinations of *Apc* loss and activation of NF-κB induce de-differentiation and drive tumorigenesis from villi [124]. A similar outcome is observed upon loss of TGFβ, through inactivation of the TGFβ type 1 receptor in animals deficient for *Apc* and carrying a constitutively active *Kras* mutation [125]. Further studies to better define the mechanisms through which Wnt signalling drives intestinal tumourigenesis are vital to identify novel therapeutic targets for CRC.

## 7. Wnt Signalling in Intestinal Hyperplasia and Ageing in *Drosophila*

Multiple studies have shown that the *Drosophila* midgut undergoes age-related dysfunction of the intestinal epithelium, which is characterized by excessive ISC proliferation and aberrant differentiation [126,127,128,129]. ISC proliferation dictates the global wellbeing of the organism and animal lifespan [130]. Midgut epithelial *Wg* expression is induced upon ageing and drives age-dependent ISC hyperproliferation through activation of its target dMyc within ISCs [87] (Figure 2iii). Critically, partial knockdown of Wg or Myc prevents age-dependent intestinal hyperplasia without disrupting ISC homeostasis [87], highlighting the potential benefits to the organism of maintaining controlled levels of Wnt signalling activation in the intestine. Interestingly, a recent report reveals differences in the way Wnt signalling is regulated in the ageing mouse and fly intestine. Unlike in flies, the ageing mouse intestinal epithelium displays a reduced regenerative potential due to a decline in canonical Wnt signalling [90] (Figure 1iv). It therefore appears that the ageing fly intestine is more similar to ageing haematopoietic stem cells and the skin epidermis, which are also characterized by exacerbated Wnt signaling [98,131,132], than to the ageing mouse intestine.

The adult *Drosophila* midgut has been successfully used to model various aspects of colorectal-cancer-like hyperplasia. Over activation of Wnt signalling through overexpression of *wg*, activated β-catenin or loss of *Drosophila Apc* leads to increased ISC proliferation and epithelial hyperplasia [86,133,134,135] (Figure 2iv). Downregulation of dMyc or overexpression of dominant negative Tcf, suppresses intestinal hyperplasia after *Apc* loss [133,135], suggesting the involvement of the canonical pathway in this process. Recent work has revealed that two conserved suppressors of *Drosophila Apc1*, earthbound (Ebd) and erect wing (Ewg) cooperate with β-catenin and Tcf to promote target gene activation and intestinal hyperplasia following loss of *Apc1* [97] (Figure 2iv). Ebd is known to physically associate with and promote the formation and stability of the β-catenin–Tcf complex and the recruitment of β-catenin to the chromatin [136]. Ewg is a DNA binding transcriptional activator that shares DNA binding specificity with the human nuclear respiratory factor-1 (Nrf-1) [137]. The potential role Nrf1, in mammalian Wnt signalling merits future investigation. Interestingly, Jerky (also known Jrk or Jh8), the human homolog of Ebd is detected at high levels in colon carcinoma and it is associated with increased nuclear β-catenin and the overexpression of Wnt target genes in human colorectal tumors [138].

Pathways mediating intestinal hyperproliferation downstream of *Apc* loss in *Drosophila* also include the EGFR/MAPK and JAK/STAT signalling. Wg signalling regulates ISC proliferation by inducing the production of ligands of the EGFR and JAK/STAT pathways in EBs and ECs respectively [135]. This paracrine EGFR and JAK/STAT signalling crosstalk mediates intestinal hyperproliferation following *Wg* overexpression and *Apc1* loss. Moreover, Wnt signalling activates the non-receptor tyrosine kinase c-Src (Src) in vivo [119]. Src drives tumourigenesis upon *Apc* loss in the adult fly midgut through ISC upregulation of EGFR and JAK/STAT signalling [119] (Figure 2iv). Lastly, as in mammals [120,121], Hippo signaling also mediates tumourigenesis of *Apc* deficient cells (*Apc*^−/−^) in the adult fly midgut [139]. Interestingly, the work in *Drosophila* reveals tumor–host-cell competition as an important determinant in the expansion of *Apc*^−/−^ cells, which appears to involve activation of the c-Jun *N*-terminal Kinase (JNK) and the Hippo signalling transcription factor Yorkie (Yki) within *Apc*^−/^^−^ cells, and apoptosis of the surrounding wild type cells [139] (Figure 2iv). Further investigation of the role of Wg signalling in cell competition within the *Drosophila* midgut could provide a new understanding on the pathology of *Apc* driven tumourigenesis in the intestine.

Oncogenic cooperation in CRC has also been modeled in *Drosophila*. Cooperation between loss of *Apc* and hyperactivation of *ras* (*Apc*^−/−^, *ras^V12^*), which characterizes malignant stages of human colorectal tumours [140,141], drives the progression of *Apc* mutant intestinal tumors and the activation of *Apc*^−/−^, *ras^V12^* specific transcriptional targets in the *Drosophila* adult midgut [142,143]. This paradigm should not only provide an excellent model to analyze the genetic events involved in malignant tumor progression, but may also represent an attractive system to identify processes specific to such genetic combinations and to test therapeutic agents. In fact, chemical compounds have been successfully used in complex *Drosophila* models bearing combinations of various ‘CRC-like’ oncogenic mutations that generate invasive intestinal tumours derived from differentiated hindgut cells, the functional equivalent of the mammalian colon [144]. Further use of the above-described paradigms is likely to provide new insights into the functional molecular networks driving various stages of CRC, which may contribute to the design of personalized therapeutics for the disease.

## 8. Conclusions and Perspectives

Many interesting questions remain to be addressed regarding the role of Wnt signalling in the intestine and, in particular, in ISC function. Understanding the role of ‘regeneration specific’ Wnt ligands, which are redundant for basal tissue homeostasis, is one of them. Studies in both mice and *Drosophila* have evidenced the existence of damage-inducible Wnt stem cell niches that are specifically needed to drive the acute proliferative response of ISCs following injury, but are dispensable for homeostatic tissue self-renewal [71,87]. Intestinal regeneration shares many molecular features of tumorigenesis [116,119]. Identification of the mechanisms activated by damage/stress-inducible sources of Wnt ligands might represent an excellent therapeutic window for the targeting of Wnt-driven intestinal hyperplasia while preserving organismal health.

Another interesting aspect of Wnt signalling in the intestine relates to the role of short range signalling in the system. Pioneer work in *Drosophila* revealed that restriction of *Wg* secretion through cell membrane tethering maintains cell growth functions of the ligand in the wing disc [145]. Later work in the mammalian intestine presented evidence for short-range Wg signalling in stem cell proliferation through membrane-tethered *Wnt3* [89]. The extent of developmental and adult intestinal-specific functions of such short range signalling remains largely unexplored. *Drosophila* is likely to provide invaluable answers to this and other unexplored aspects of the regulation of intestinal health and disease by Wnt signalling.

New, sophisticated mammalian CRC models are being successfully created through the use of novel technologies such as CRISPR/Cas9. This includes the generation of mouse models carrying multiple gene mutations [146], metastatic CRC models [147,148,149,150], tools to trace cancer stem cells in vivo [151] and complex gene editing within cultured intestinal organoids [152,153]. It will only be a matter of time before the conservation of intricate molecular networks identified in *Drosophila* can be assessed in such powerful mammalian paradigms.

## Figures and Tables

**Table 1:** List of fly and mammalian Wnts and their expression and function within the intestine. Known fly and mammalian Wnt ligand genes and their expression status in the intestine as determined by FlyGut-seq and NCBI, respectively. Reported intestinal function of Wnts is referenced.

Species	Wnt Type	Vertebrate Ortholog	Intestinal Expression	Main Intestinal Function
*Drosophila Melanogaster*	Wg	Wnt1	+	Required for intestinal homeostasis, regeneration and ageing [86,87]
	Dwnt2	Wnt7	-	
	Dwnt3/5	Wnt5	-	
	Dwnt4	Wnt9	+	Unknown
	Dwnt6	Wnt6	-	
	WntD/Dwnt8	-	-	
	Dwnt10	Wnt10	-	
*Mus Musculus*	Wnt1		-	
	Wnt2		+	Intestinal development [88]
	Wnt2b/13		+	Secreted by sub epithelial and mesenchymal cells and essential for gut homeostasis [62]
	Wnt3		+	Secreted from Paneth cells and essential for stem cell maintenance [37,89]
				Reduced expression in ageing ISCs [90]
	Wnt3a		-	-
	Wnt4		+	Intestinal development [88]
	Wnt5a		+	Intestinal development [88]
				Intestinal elongation [91]
				Stromal macrophage induced expression upon regeneration [73]
				Colonic crypt regeneration [72]
	Wnt5b		+	Intestinal development [88]
	Wnt6		+	Intestinal development [88]
				Transcriptionally upregulated upon damage by irradiation in crypt epithelial cells [92]
	Wnt7a		-	-
	Wnt7b		-	-
	Wnt8a		+	Intestinal development [88]
	Wnt8b		-	-
	Wnt9a		+	Suppressor of proliferation in CRC [93]
	Wnt9b		+	Expressed in Paneth cells [82]
	Wnt10a		+	Unknown
	Wnt10b		-	-
	Wnt11		+	Intestinal development [88]
				Expressed in adult intestine [94]
	Wnt16		Low	Unknown
